# A New Washable UHF RFID Tag: Design, Fabrication, and Assessment

**DOI:** 10.3390/s20123451

**Published:** 2020-06-18

**Authors:** Aurelian Moraru, Corneliu Ursachi, Elena Helerea

**Affiliations:** 1 Multidisciplinary Doctorate School, Transilvania University of Brasov, Promar Textile Industries Brasov, 500036 Brasov, Romania; amoraru@promar.ro; 2 Electrical Engineering and Applied Physics Department, Transilvania University of Brasov, Promar Textile Industries Brasov, 500036 Brasov, Romania; corneliu.ursachi@unitbv.ro; 3 Electrical Engineering and Applied Physics Department, Transilvania University of Brasov, 500036 Brasov, Romania

**Keywords:** RFID tag, passive washable tag, UHF antenna, RFID transponder, textile products

## Abstract

This paper deals with the design and fabrication of durable radio frequency identification (RFID) passive tag with inductive coupling, operating at ultra-high frequencies, dedicated to the identification and monitoring of professional textile products. A reliable architecture for the tag transponder is proposed, featuring a minimal number of galvanic contacts: The two pins of the integrated circuit are connected to the terminals of the inductive coupling loop by using surface mount technology welding. The transponder is encapsulated with an electrically insulating material which is waterproof and resistant to mechanical, thermal, and chemical stress. The antenna is inductively coupled to the transponder through a double loop which substantially reduces the length of the tag and significantly improves the coupling factor, enabling the tag to operate at a low power level. The reliability and flexibility of the tag is obtained by using appropriate materials and manufacturing methods for the ultra-high frequency (UHF) antenna by embroidering a multifilament stainless steel wire on textile support. The washing cycle tests have validated the applicability of this flexible and washable RFID tag, and its electromagnetic performance was experimentally assessed in an independent laboratory.

## 1. Introduction

The radio frequency identification (RFID) is one of the wireless technologies that implements the internet of things (IoT) concept and is now successfully applied to assure the traceability and monitoring of objects [1,2]. A generic RFID system consists of a RFID tag fixed on the object it identifies, an RFID reader, and a software application. The reader sends a radio signal to the RFID tag, which, after receiving the signal, responds with a signal containing the tag identifier and other data regarding the identified object [3,4,5].

Each RFID tag contains an integrated circuit and an RF antenna. In the memory of the RFID-integrated circuit, a unique electronic product code (EPC) from 92 to 496 bits can be written. Thus, by attaching the RFID tag, each object is assigned a unique identity. This ability to uniquely identify any element of a class of entities and the huge number of identities that can be attributed constitute jointly the conceptual strength of the RFID domain. The ultra-high frequency (UHF) band allows high communication speed at distances up to about 10 m for efficient object tracking in many applications, including localization, monitoring, and control of various items [6,7].

The success in achieving RFID-integrated circuits and software development has led to lower prices and allowed a significant increase in the performance of RFID systems. As a result, RFID systems are widely used in logistics, defense, space, health, and other applications [8,9,10].

The demand for such unique identification systems is also high for the group of professional textile products: Textiles used in the hotel industry, clothing used as work uniforms in the food and processing industries, textiles used in medical and hospital units, etc., which go through several washing cycles in industrial laundries [11,12,13].

The allocation of RFID codes, unique to each textile item that is sanitized in industrial laundries, could solve multiple problems in the process control of the internal and external circulation chain of professional textile products.

However, the application of RFID tags to textiles raises several problems:

- Reading a large number of tags;
- Durability of the tags in the harsh laundry environments;
- Reading errors.

Other requirements are: Tags should be low-cost, flexible, easy-to-fabricate, and comfortable for portable textile items [14,15].

Research reported in subject literature relates to the development of UHF RFID passive tags, but mostly in terms of the tags applied to tracking food, animals, and metallic components, having as support, plastics, paper, metal, and ceramics. There is less research on the development of UHF RFID tags for wearable and washable textiles. Improvements were mainly achieved in designing the UHF antenna and making use of various materials and technologies.

UHF antennas made with electrically conductive paint, deposited on different substrates of paper, plastic, metal, or fabric, are studied in [16,17]. A solution of the RFID tag for washable objects is proposed in [18], in which the UHF antenna is embedded in the garment by means of embroidery of a conductor wire. A recent paper [19] proposes RFID antenna solutions for wearable articles, by using several materials: Commercially available electro-textile, conductive thread, or conducting paint. The textile substrate is 100% cotton fabric. The tag is fully coated with a protective encapsulate. A flexible wearable tag is proposed in [20], where the antenna wire is built of a conductive fabric composite.

Some studies are related to improving the durability of the RFID passive tag to washing cycles [11,14,15,18,21]. The effect of moisture and washing on the performance of this type of tag is also studied [22]. It was shown that repeated washing of the tag has led to a significant reduction in the RFID tag read range. It turned out that protective coating is needed, and should be flexible, durable, and hydrophobic. The effects of thermal stress on the reliability of passive RFID tags were studied in [23], with accelerated life tests.

Reliability analysis of UHF RFID tags under long-term mechanical and thermal cycling is also reported in [16,19,24,25,26]. However, not all these solutions are viable in order to ensure the reliability and robustness of the RFID tags for professional textiles in industrial laundries. It is concluded that because of the aggressive environments, not many applications have been developed, and new research should be done for developing a passive high-performance UHF tag, designed to work in aggressive industrial environments.

This paper deals with the theoretical and experimental investigations into the design and realization of a new UHF RFID passive tag prototype capable of operating in the harsh environment of textile laundry, which can be used to control the processes on the internal and external circulation chain of professional textile articles/products.

The paper is organized as follows. Section 2 contains a comparative analysis of the two architecture solutions for RFID passive tags, namely with an adaptive impedance structure and with an inductive coupling loop, and introduces the conceptual design elements of a washable UHF RFID passive tag. Section 3 describes the novel development approach of the new RFID UHF passive tag for professional textiles with an encapsulated RFID transponder, inductively coupled to the UHF antenna, and the research work undertaken for UHF RFID passive tag prototyping and performance assessment. Section 4 and Section 5 cover the discussion and the conclusions of the study, respectively.

## 2. Materials and Conceptual Design

The following steps were taken towards the development of the conceptual design of the new washable passive UHF RFID tag: (i) Establishing an appropriate tag architecture, (ii) the design of the RFID transponder, (iii) the design of the UHF antenna, and (iv) the choice of suitable materials for tag components prototyping.

### 2.1. Analysis of the UHF RFID Passive Tags Architectures

**Figure 1:**
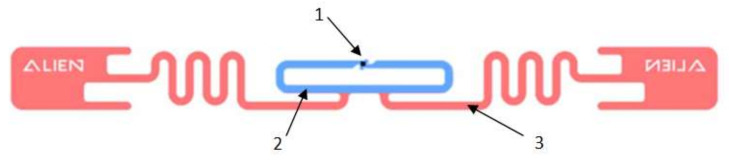
Ultra-high frequency (UHF) radio frequency identification (RFID) passive tag architecture with impedance adaptation structure [27].

Historically, the first architecture used in designing the UHF RFID passive tags for harsh industrial environments was the one in which the UHF antenna integrates an adaptative impedance structure, galvanically coupled to the integrated circuit [3,4]. An example is shown in Figure 1 that highlights a typical passive RFID UHF tag architecture with impedance adaptation structure, consisting of: Integrated circuit (1), impedance adaptation structure (2), and UHF dipole antenna (3).

Regarding the role of the adaptive impedance structure, this matches the UHF antenna impedance to that of the RFID-integrated circuit in the UHF RFID frequency band, adding an inductive reactance to that of the UHF antenna to compensate the capacitive reactance of the integrated circuit, thus achieving the resonance condition of the UHF RFID passive tag’s entire electric circuit [3]. Its constructive structure, in which the tiny pins of the integrated circuit are galvanically connected to the adaptive impedance structure that has much larger dimensions, has proven unreliable for RFID tags working in the severe environment of industrial laundries: The tiny electrical contacts break down after several washing cycles. Therefore, in the case of passive UHF RFID tags for professional textiles that are sanitized in industrial laundries, this architecture has been replaced with one based on inductive coupling between antenna and integrated circuit.

**Figure 2:**
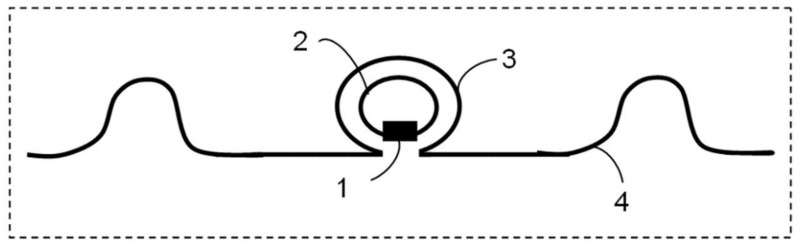
UHF RFID passive tag architecture with inductive coupling.

In the case of UHF RFID passive tags with inductive coupling, a compact transponder containing the integrated circuit is inductively coupled with the UHF antenna (Figure 2).

**Figure 3:**
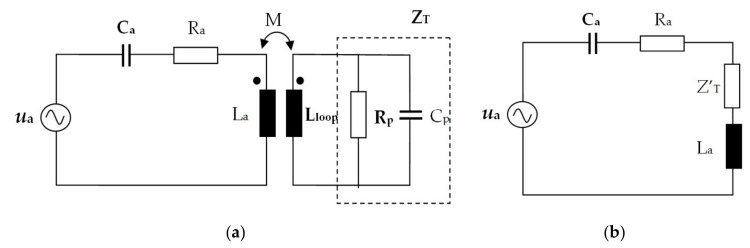
Electrical schemes for UHF RFID passive tag: (**a**) The circuits of antenna and transponder, inductively coupled; (**b**) the circuit of antenna with the load impedance introduced by the inductive transponder–antenna coupling.

The transponder consists of the RFID-integrated circuit (1) and the transponder inductive coupling loop (2). The tag antenna consisting of a meandered radiating element (4) contains a spiral shaped coupling loop (3). The equivalent electric scheme in Figure 3 highlights the inductive coupling between the UHF antenna and the RFID transponder, given by mutual inductance *M*.

The integrated circuit is modeled with the impedance *Z_T_* consisting of the parallel *R_p_* and *C_p_* components. The antenna is modeled with capacitance *C_a_*, radiation resistance *R_a_*, and inductance *L_a_*. The electromotive force induced in the transponder circuit through the inductive coupling ensures the transfer of energy and information. The circuit shown in Figure 3b allows calculating the load impedance of the circuit.

The voltage equation for the equivalent circuit from Figure 3b is:

(1) $$U_{a}=\left[R_{a}+j\left(\omega L_{a}-\frac{1}{\omega C_{a}}\right)+j\omega M\cdot \frac{j\omega M}{j\omega L_{loop}+Z_{T}}\right]\cdot I_{1}$$

where mutual inductance is given by the relation:

(2) $$M=k\cdot \sqrt{L_{a}\cdot L_{loop}}$$

which allows for the deduction of the load impedance of the transponder included in the antenna circuit due to the inductive transponder–antenna coupling:

(3) $$Z_{T}^{\prime }=\frac{\omega^{2}k^{2}L_{a}\;L_{loop}}{j\omega L_{loop}+Z_{T}}$$

The load impedance depends on the square of the frequency *f* and of the coupling factor *k*, on the antenna inductance *L_a_* and of transponder loop inductance *L_loop_*, and on the parameters of the integrated circuit. This analysis shows that by an adequate design of the antenna geometry and of the inductive coupling, the appropriate values of the parameters *R_a_*, *C_a_*, *L_a_*, and *Z_T_* are obtained, which ensure the resonance of the equivalent circuit of the tag. The read range *d_r_* of an RFID tag may be estimated using Frii’s transmission Equation (4) as:

(4) $$d_{r}=\frac{\lambda }{4\pi }\cdot \sqrt{\frac{P_{r}G_{r}G_{t}\tau }{P_{ICm}}}$$

where *λ* is the wavelength, *P_r_* is the power delivered by the RFID reader, *G_r_* is the gain of the reader antenna, *G_t_* is the gain of the tag antenna, *P_ICm_* is the minimum power required to activate the RFID-integrated circuit, and the power transmission coefficient *τ* is given by:

(5) $$\tau =\frac{4R_{\mathrm{IC}}R_{a}}{{\left|Z_{\mathrm{IC}}+Z_{a}\right|}^{2}}$$

where in *Z_IC_* = *R_IC_* + *jX_IC_* denotes impedance of the RFID-integrated circuit and *Z_a_* = *R_a_* + *jX_a_* denotes impedance of the antenna structure.

A good read range in the case of the tag with this architecture can be obtained by designing an adequate geometry of the antenna, achieving an efficient inductive antenna–transponder coupling, and by using an integrated circuit with high sensitivity. 

### 2.2. Conceptual Design of a New Washable UHF RFID Passive Tag

For the sizing of the UHF RFID tags for harsh environments, the authors elaborated on various constructive models of tags with inductive coupling. The advantage of this architecture, given by eliminating the galvanic connection between the integrated circuit pins and the adaptive impedance structure, was taken into account; the risk of breaking this connection was therefore eliminated and thus the tag becomes more resistant to the harsh working environment. In the patent reported in [28], the inductive coupling loop is arranged on the whole circumference of the encapsulated transponder, in [29] on about 5/6 of the circumference, and in the project [30] on 1/2 of the circumference. In all these models, to reduce the length of the tag, the UHF antenna’s dipole arms have loops and meanders. These solutions offer some advantages, such as: (i) Resistance to mechanical, thermal, and chemical stresses specific to industrial laundries by eliminating the galvanic connection between the antenna and the integrated circuit, (ii) increased flexibility given by the small size of the encapsulated transponder. 

However, such models do not directly ensure the reliability required for passive UHF tags for textile items which require repeated washing. Because UHF antenna plays an important role in RFID systems, much research has focused on optimizing the tag’s UHF antenna structure [23] or choice of materials [16,17,18,19,20].

Research related to the improvement of electromagnetic characteristics of the RFID passive tags (resonant frequency, the impedance) has been extended by taking into consideration the specific character of the targeted object (shape, size, geometry, composition) and the object operating environment [23,31,32,33]. The impact of the electrical properties of the substrate material on the performance of the UHF RFID tags is covered in [16]: The dielectric loss factor affects the efficiency. In designing the new passive UHF RFID tag for professional textiles, the authors took into account the specific operational requirements and the constraints related to the working environment, the integrated circuit parameters, and the UHF antenna. These requirements were customized for the washable RFID tag: The tag must be of the UHF type, with reasonable size and with an encapsulated RFID transponder inductively coupled to a UHF antenna, eliminating the galvanic connection between the integrated circuit and tag antenna [30,34].

#### 2.2.1. Conceptual Design of the New UHF RFID Transponder

The choice of the integrated circuit (IC) is made according to the requirements imposed by the functionality of the passive UHF RFID tag and the application for which they are intended. An essential parameter is the sensitivity of the IC, defined by the minimum power required to activate the RFID-integrated circuit, for communication *P*_com(min)_, and for programming *P*_P(min)_. In this regard, the evolution of RFID-integrated circuit parameters was taken into account. From powers of 1 mW in 2001, units of µW in 2010 were reached. Since 2003, RFID-integrated circuits with sensitivities of 16 µW have been used, in 2008 of 2.7 µW and below 1 µW at the current time. In 10 years, the sensitivity of RFID-integrated circuits has increased 1000 times [35]. Currently, the UHF RFID-integrated circuits of the passive tags on the market have sensitivities of the order of 10 µW (−20 dBm).

For harsh environments, ICs that can withstand high temperatures and repeated temperature variations must be chosen. For example, Table 1 shows the operating conditions and electrical parameters for two RFID reference ICs that could operate in harsh environments. They are ICs with good electromagnetic performance in the SOT 323 package, resistant to thermal stress.

Higgs4 was chosen due to its much better performance in terms of sensitivity. Regarding the operating conditions, the Higgs4 performance to thermal stress is relatively good and can be increased through an additional encapsulation protection.

For the conceptual design of the inductive coupling loop of the transponder, the functions of the inductive coupling loop were taken into account: (i) To perform the resonance in the electrical circuit of the transponder, (ii) to communicate by inductive coupling with the UHF RFID antenna of the passive tag. 

Once the IC is chosen, the values of resistance *R_p_* and capacitance *C_p_* of the equivalent input parallel RC circuit of are known from the datasheet [32]. In transponder design, it is useful to know the equivalent input series resistance *R_s_* and capacitance *C_s_*, calculated with relations:

(6) $$R_{s}=R_{p}\cdot \frac{1}{1+{\left(\omega R_{p}C_{p}\right)}^{2}};\;C_{s}=C_{p}\cdot \frac{1+{\left(\omega R_{p}C_{p}\right)}^{2}}{{\left(\omega R_{p}C_{p}\right)}^{2}}$$

For example, for Higgs™ 4 SOT integrated circuit (Table 1), at 915 MHz, with given parallel equivalent resistance *R_p_* = 1.8 kΩ and capacity *C_p_* = 0.95 pF, the series equivalent parameters are: *R_s_* = 18.45 Ω and *C_s_* = 0.959 pF. The inductance of the loop *L_loop_* is obtained with the resonance condition that is established between the loop and the equivalent serial capacity *C_s_* of the integrated circuit:

(7) $$L_{loop}=\frac{1}{{\left(2\pi f_{0}\right)}^{2}C_{s}}$$

Thus, for the resonance frequency *f_o_* = 915 MHz, in relation to Equation (7), an inductive coupling inductance of *L_loop_* = 31 nH is required. It should be noted that in order to obtain this inductance value, a single circular loop should have a diameter of 12 mm [36], which would lead to a considerable size of the transponder, and consequently, of the tag. However, the market demands small tags. 

The challenge therefore is to achieve an inductance of a certain value, on a surface, as small as possible, with a minimum number of galvanic contacts between the RFID IC and the inductive coupling loop, based on the simplest implementation solution. 

**Figure 4:**
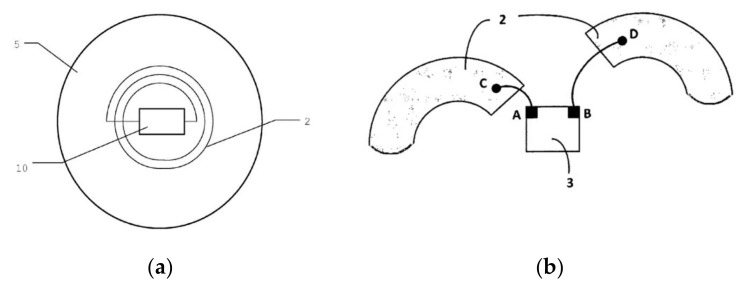
The inductive coupling loop with spiral geometry: (**a**) General view; (**b**) detail of electric connections.

A spiral geometry for the inductive coupling loop was adopted in some cases (Figure 4) [30], where a spiral loop surrounds the UHF RFID-integrated circuit.

The number of galvanic connections here is four (A, B, C, D) (Figure 4b). The welds are very fine and sensitive to repeated temperature variations. So, in this case, there are two vulnerabilities: The number of galvanic connections and the sensitivity to repeated temperature variations.

**Figure 5:**
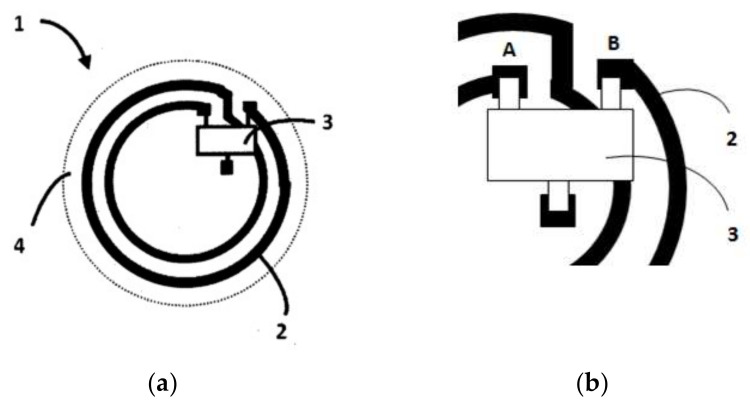
The inductive coupling loop of transponder with circular geometry: (**a**) General view; (**b**) detail of connections.

The novel geometry proposed by the authors is a flat circular geometry for the inductive loop (Figure 5a) of the transponder (1), made of two concentric turns (2) of about 5 mm in diameter, surrounding the integrated circuit (3), the ends being connected to the IC pins on a printed circuit board substrate (4). 

Specific to the proposed geometry is the preservation of the circular shape of the traces, the transition from the circle with the smaller radius to the one with the larger radius being realized as in Figure 5b [34]. The integrated circuit with the SOT323 package was chosen, which has a distance between points A and B large enough to permit the passing of the trace between them. The distance between the inner starting point and the outer ending point of the spiral becomes the distance between the terminals of IC. So, straps are not necessary anymore, nor are VIAs needed, thus the transponder can be achieved on a single size PCB.

This design allows to have only two galvanic connections, the minimum possible. Also, the two galvanic connections become bigger and more reliable than those of Figure 4b, and through their dimensions they become more reliable against temperature stress. 

#### 2.2.2. Conceptual Design of a New UHF Antenna Geometry

**Figure 6:**
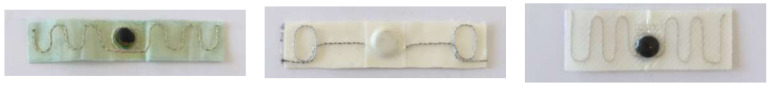
The geometries of UHF antennas for some UHF RFID passive tags.

The design of UHF antenna for washable RFID tag involves establishing an appropriate geometry that ensures the functionality of the tag [37]. The representative tags on the market have the UHF antennas with a simple inductive loop that closely surrounds the transponder (Figure 6).

Different geometries of the lobes of the UHF dipole antenna were used: Sinusoidal geometry with different shape factors and geometry with lobes in the form of closed curves at the tag’s ends. 

**Figure 7:**
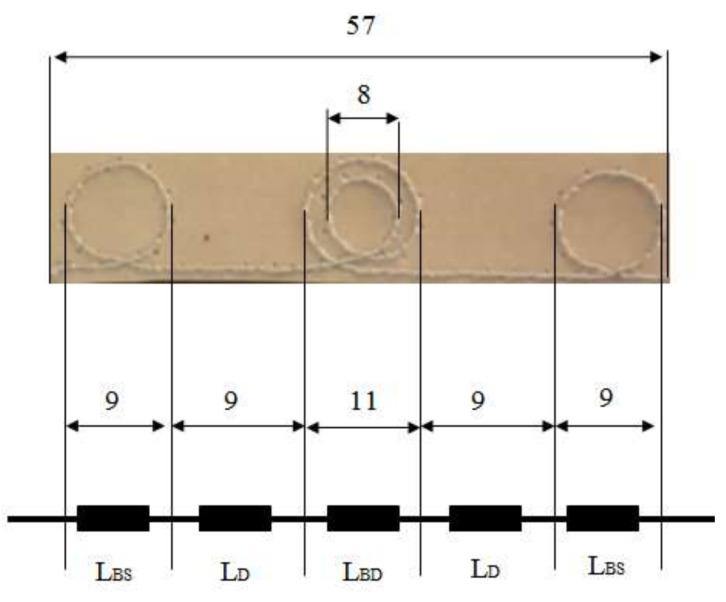
Mode of segmentation for calculating UHF antenna inductance.

In order to reduce the tag size, the authors performed experimental research and established an antenna geometry having a double coupling loop in the central part and two single loops at its ends. [38]. For the inductance calculation, the conductive structure of the UHF antenna is divided into segments with geometries for which there are inductance calculation relations. Figure 7 shows the segmentation mode in the case of the proposed double inductive coupling loop UHF antenna.

The conductive structure of the UHF antenna consists of a double loop in the central area, two single loops at the ends, and two rectilinear segments. The conductor of antenna wire has diameter *D* = 0.25 mm. The total inductance is the sum of the inductances of the component segments. In this approach, the mutual inductances between the segments of the conductive structure are neglected. To perform the calculations, the double loop in the central area is equivalent to a solenoid with two turns of average diameter *D_BD_* = 9.5 mm, and the loops at the ends of the antenna are considered solenoids with one turn and diameter *D_BS_* = 9 mm. With the software application [39], the inductance value of the UHF antenna of *L_a_* = 145.3 nH was obtained. The radiation resistance *R_a_* of proposed antenna is calculated with:

(8) $$Ra=\frac{l_{a}^{2}}{l_{r}^{2}}\cdot Rr$$

where *l_a_* is antenna length, *l_r_* is *λ*/2 dipole length (*l_r_* = 173 mm for *f* = 900 MHz), *R_r_* is radiation resistance for *λ*/2 dipole (*R_r_* = 65 Ω [3]). For a length of antenna *l_a_* = 57 mm, the radiation resistance is *R_a_* = 7.57 Ω.

It should be mentioned that by reducing the length of the antenna, the capacitance and inductance of the antenna decrease, which leads to an increase in the capacitive character of the UHF antenna. The geometry proposed for the antenna ensured the creation of an inductance that effectively compensates the capacitive character of the antenna, so that the resonance takes place at the frequency of 900 MHz, in the regulated RFID domain.

## 3. Results Regarding Tag Fabrication and Assessment

### 3.1. Models of UHF RFID Passive Tags

**Figure 8:**
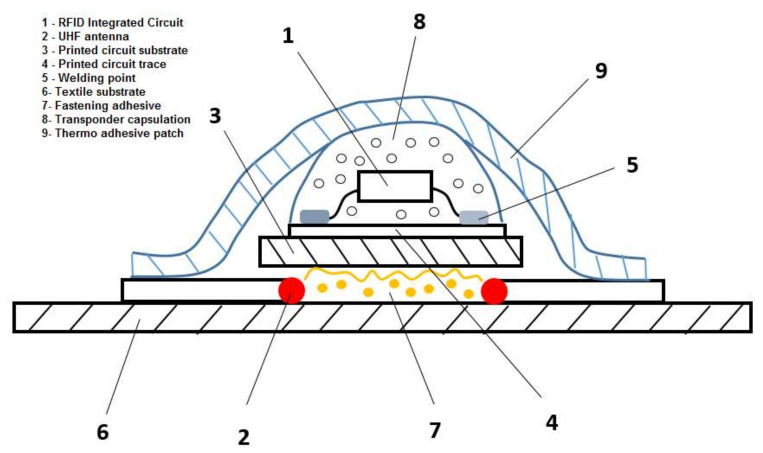
Detail with the components of the proposed passive UHF RFID tag.

The authors conceived and designed several models/constructive solutions of UHF RFID passive tags with inductive coupling loop. Figure 8 shows a detail of the proposed tag structure: RFID-integrated circuit (1), UHF antenna (2), printed circuit substrate (3), printed circuit (4), pin welding point of the integrated circuit in surface mount technology (5), textile support (6), adhesive for fastening the encapsulated transponder (7), protective cover of the transponder (8), thermo-adhesive textile tape for fixing the transponder on the UHF antenna (9).

The models implemented have the substrate support of textile material with a mixed composition 50% cotton and 50% polyester (number of fine yarns 200), with the outer surface impregnated with a thermo-adhesive film, for fixing the tag on the textile article. The transponder includes the integrated circuit (IEC 18000-6C standard) and the inductive coupling loop, designed and prototyped by the authors on PCB support, in surface mounting technology. The assembly, cylindrical in size (*D* = 5–7 mm, *h* = 1–2 mm), is protected by epoxy resin encapsulation.

The UHF antennas were implemented with different technologies and materials; thus, four passive UHF RFID tag models with inductive coupling for textile articles have been experimentally implemented/fabricated. Variants of passive RFID UHF tag models are shown in Table 2. 

**Figure 9:**
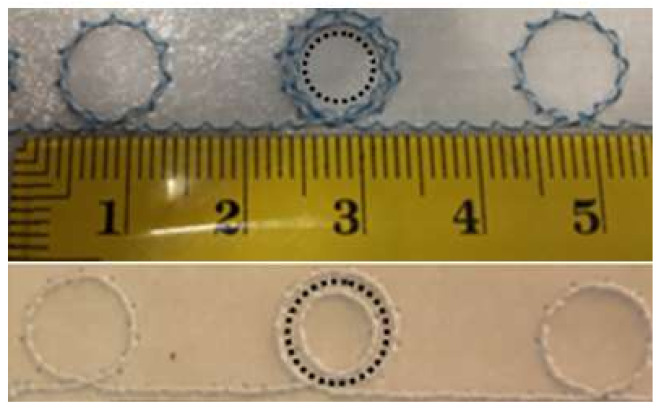
M4 washable RFID tag model with embroidered UHF antenna and transponder arranged inside the circular double loop of the antenna.

Figure 9 shows the borderline of the M4 washable UHF RFID tag with embroidered UHF antenna and transponder arranged inside the small circular double loop.

All these models have as a characteristic the special geometry of UHF antenna: In the central area it has a double inductive coupling loop, and at the ends, simple loops. This new geometry, by the presence of the double loop, significantly reduces the length of the UHF RFID passive tag. 

### 3.2. UHF RFID Passive Tag Prototyping and Performance Assessment

#### 3.2.1. The Washing Cycle Testing of the Model Tags

In order to establish the most adequate passive tag solution to withstand the harsh environment in industrial laundries, preliminary tests were performed on sets of samples prototyped based on the four models described in Table 2. Preliminary test planning was established, taking into account current research on cyclic wash test procedures [14,16,18] and methods for determining component reliability [22,23].

The parameters corresponding to industrial wash cyclic test are described in Table 3.

The objective of the test was to assess the durability of the four tag models, subjected to the conditions corresponding to the industrial washing process, described in Table 3. Each batch of 50 tags, built according to the models described in Table 2, was subjected to the repeated washing test in an industrial rotary washing machine. After each cycle, the visual examinations of the tags were made and their RFID performance was measured. The initial measurement of RFID performance demonstrated that all the tag models achieved a very similar initial maximum read range, i.e., an average of *d_r_* = 9.5 m, with less than 2% relative standard deviation. This demonstrates the reproducibility of the proposed fabrication approach. 

After each washing cycle, the RFID performance of the tags was measured, with the reader antenna located 2 m away from the tag batch. For a reader transmitting power *P_r_* = 500 mW, the reading speed and the number of successful readings were registered. Table 4 presents the performance of passive RFID UHF tag models after testing at the limit number of wash cycles.

The visual examination of the tag models after each wash cycle has shown a different durability at cyclical wash: Conductive paint of model M1 begins to crack, copper wire of model M2 begins to deform and bend, and conductive particles of the textile thread of the M3 model begin to be dissipated in water. After 30 cycles, models M1, M2, and M3 become non-functional. For these models, after about 30 consecutive cycles of industrial washing, the RFID performance decreased drastically, the electrical characteristics of the antenna degraded, and the tags were no longer read by the reader.

The behavior at cyclic washing stresses for the M4 model is different: The stainless steel multi-wire is flexible and also resistant to mechanical and thermal stresses, and also to the action of detergents. The tags of this model remained functional until about 400 washing cycles. Thus, this assessment procedure showed that the M4 model of RFID tag is the most suitable prototype to represent the proposed new UHF RFID tag solution, based on highest durability.

#### 3.2.2. Bending Testing of M4 Model Tags

The purpose of the test was to choose the antenna construction that best withstands the bending stress. The tag samples were built with the same transponder structure corresponding to the M4 tag model, and with the meandered antennas made of two types of multifilament stainless steel wires, one with 4 filaments of 34 µm diameters and the second with 275 filaments of 12 µm diameters. Mechanical bending tests shown that after 30–50 repeated bending, the UHF antenna made of a 4-filament multifilament wire gave in and had poor reading performance. 

Mechanical bending tests highlight the superior reliability of the tag with UHF antenna made of multifilament wire with 275 filaments. The advantages are: Flexibility and resistance to bending; elastic behavior (returns to the original shape after elbows in washing mode); allows the realization of routes with small radii of curvature; good resistance to aggressive chemicals present in the technological process of industrial laundry, which do not corrode. However, there are also disadvantages: Antennas are more rigid than those of previous solutions, have a lower electrical conductivity, but this does not affect the electromagnetic performance of the antenna. 

Thus, taking into account all aspects, it was established that the model M4 of tag with UHF embroidered antenna made with multifilament wire with 275 stainless steel filaments satisfies the washing environment requirements. This model can represent the prototype UHF RFID tag, called Dac100. 

### 3.3. Electromagnetic Performance Validation of the Dac100 Prototype

The performance of the Dac100 tags was validated in the Voyantic Laboratory [40], equipped with facilities and measuring equipment that allow measurements in accordance with RFID and measurement standards in the field of radio frequency. The measurements were performed in an anechoic chamber, in which the reader–tag communication is protected from reflections and electromagnetic interferences specific to the industrial environment. The frequency band in which measurements were made was 800–1000 MHz. Comparative measurements were made for a batch of Dac100-type tags, having a UHF antenna with a double inductive coupling loop and other types of tags, representative of the market [41,42,43,44], and having the UHF antenna with single inductive coupling loop. A description of UHF RFID tag variants is given in Table 5.

**Figure 10:**
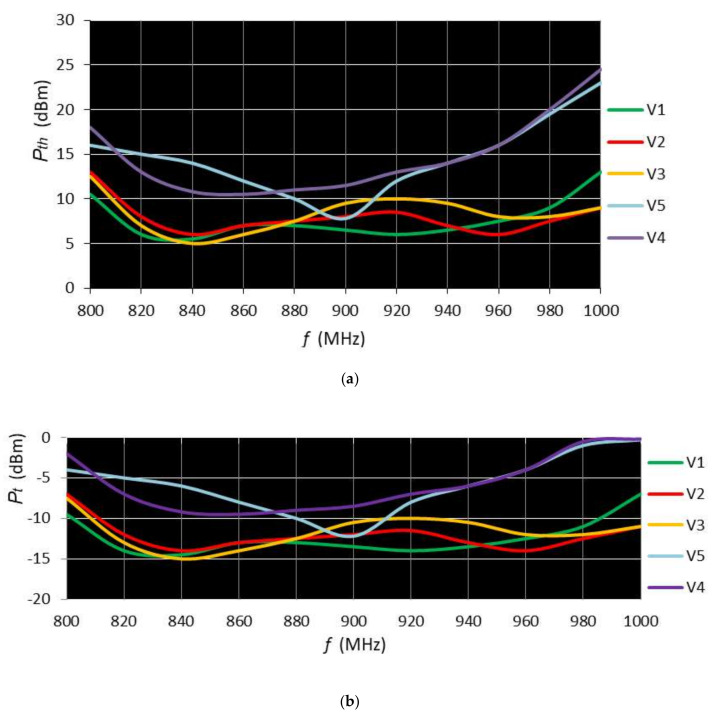
The power of the tags in 800–1000 MHz frequency range: (**a**) Threshold power *P_th_*; (**b**) power on tag forward *P_t_*.

The assessed performance parameters were the threshold power *P_th_* and the read range *d*_r_. For the threshold power measurement, the tags were located at *d* = 1.5 m away from the reader and the power was reduced to the minimum level at which communication was possible. The graphs in Figure 10 shows the threshold power *P_th_* (Figure 10a) and the power on the tag forward *P_t_* (Figure 10b) in 800–1000 MHz frequency range.

**Figure 11:**
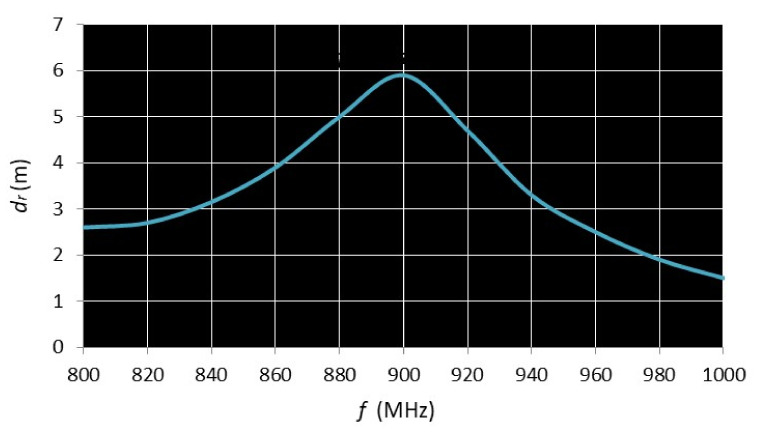
The read range *d_r_* as a function of frequency for V5 (Dac100) tag.

Another parameter for assessment of the passive RFID UHF tags is the read range as a function of frequency. For the read range measurement, the reader power was set to 1 W and the distance was increased until the communication stopped. The measurement result regarding the read range for the developed double loop tag (V5) is shown in Figure 11.

**Figure 12:**
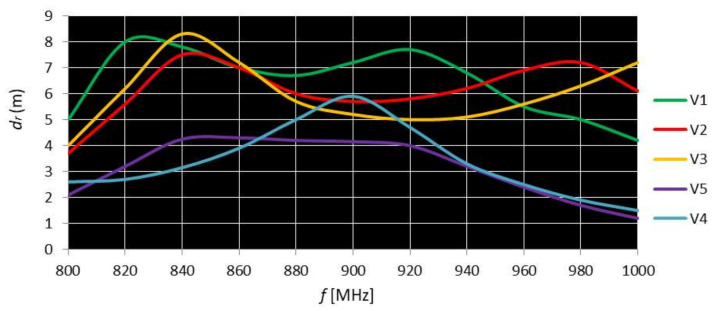
The read range as a function of frequency for double and single coupling loop tags.

Figure 12 shows the reading range as a function of frequency for the double-loop V5 tag compared to the most representative single-loop tags on the market.

## 4. Discussion

The durability in harsh environments of the four tag models was assessed using the washing cycle test, which reproduced the conditions corresponding to the industrial washing process. Based on this test and on the bending test, it was established that the model M4 of tag with UHF embroidered antenna made with multifilament wire is the most suitable UHF RFID tag prototype. Electromagnetic performance was assessed in an anechoic chamber. The graphs in Figure 10a highlight the following:

- Tags with larger sizes (V1, V2, V3) have a threshold power of about 5 dBm at 840 MHz, which is outside the regulated RFID frequency range 860–960 MHz;
- Tags with smaller dimensions (V4, V5) have a minimum threshold power of 12 dBm (V4) and 8 dBm (V5) in the frequency range of 900–915 MHz, in the middle of the regulated RFID range 860–960 MHz, which reflects an appropriate design;
- In the frequency range of 900–915 MHz, the Dac100 (V5) tag has a better minimum threshold than those of V3 and V5, the same as that of V2, and higher than that of V1, which shows the reduction in size does not lead to a significant decrease in performance.

Power on tag forward *P_t_* graphs (Figure 10b) follow the shape of transmitted power *P_th_* with a 20 dBm difference. Figure 11 shows that for the V5 tag, a maximum read range of 5.9 m is observed in the frequency range 890–900 MHz. 

The graphs in Figure 12 highlight the following:

- Tags with larger sizes (V1, V2, V3) have a maximum read range of 7.5–8.5 m in the frequency range of 820–840 MHz, outside the regulated RFID range 860–960 MHz;
- Tags with smaller dimensions (V4, V5) have a maximum read range of 4.3, respectively, 5.9 m in the frequency range of 900–915 MHz, interval in the middle of the regulated RFID domain 860–960 MHz;
- In the frequency range of 900–915 MHz, the Dac100 (V5) tag has a longer read range than those of V2, V3, V4, and a read range less than that of V1.

Table 6 summarizes the values of the threshold power *P_th_* and the read range, at the frequency of 900 MHz, for the variants of passive RFID UHF tags described in Table 5. 

Data in Table 6 confirm that V5-Dac100, the tag designed and realized in present research, has a level of performance comparable with that of the best washable UHF RFID passive tags on the market. For Dac100 having the dimension of 57 × 13 mm, the important requirement to be as short as possible was achieved, with an acceptable decrease in performance.

Experimental results showed that the RFID tag with such an embroidered antenna responds well to queries at maximum emission power, and that it responds better to queries at low powers.

## 5. Conclusions

The architecture of the UHF RFID passive tag with an encapsulated RFID transponder, inductively coupled to the double-loop UHF antenna, proposed by the authors, represents a suitable solution that meets the requirements of the harsh washing environment. 

The RFID tag transponder has two galvanic contacts to the terminals of the inductive coupling loop and is encapsulated with an electrically insulating material which is waterproof and resistant to mechanical, thermal, and chemical stress. The UHF antenna is inductively coupled to the transponder through a double loop, which substantially reduces the length of the tag and significantly improves the coupling factor, enabling the tag to operate at a low power level. 

The reliability and flexibility of the tag is achieved by using appropriate materials and manufacturing methods for the UHF antenna by embroidering a multifilament stainless steel wire on textile support. The washing cycle and bending tests validated the applicability of this washable and flexible UHF RFID passive tag. 

The electromagnetic performance, assessed in an independent laboratory, is comparable to the performance of the best washable UHF RFID passive tags on the market.

## Figures and Tables

**Table 1:** Parameters of integrated circuits for UHF RFID tags in [27] datasheets.

Symbol	Parameters	Higgs™ 3 SOT	Higgs™ 4 SOT
*T_o_*	Operating temperature (°C)	−50 … +85	−50 … +85
*f_o_*	Operating frequency (MHz)	860 … 960	840 … 960
*T_MT_*	Maximal temperature/time (°C/s)	260/60	235/60
*P*_com(min)_	Minimum RF communication power (dBm)	−17	−20.5
*P*_P(min)_	Minimum RF programming power (dBm)	−10	−17
*R_p_*	Equivalent input parallel resistance (Ω)	1500	1800
*C_p_*	Equivalent input parallel capacitance (pF)	0.85	0.95

**Table 2:** Passive RFID tag models and materials and technologies for building UHF antennas.

Model.	Materials for UHF Antenna	Technology for UHF Antenna Building & Details
M1	Electroconductive paint	Screen printing process on textile media; The length of the contour: *λ*/2 = 172 mm for *f_med_* = 866.5 MHz; the width of the contour *l* = 1 mm.
M2	Copper conductor plated with silver	Modeling of silver-plated copper wire and fixing with adhesive on textile support; The diameter of conductor is *d* = 0.25 mm; the insulation of the conductor is achieved with teflon.
M3	Electroconductive textile thread	Electroconductive textile wire modeling and gluing on textile support; The round filament with polyamide core 6.6 and 99% silver metallic coating, wire resistance is *R* = 30 Ω/m.
M4	Multifilament stainless steel wire	Modeling multifilament steel wire fixed on the textile support by embroidering; The stainless steel wire has 275 filaments with the diameter *d* = 12 μm, the insulation of the wire being made of teflon or polyester.

**Table 3:** Parameters for wash cyclic test.

Description of the Test Environment	Washing Scheme for Tag Testing
For washing process	Δ*T* = 8 °C to 90 °C, 20 s; *T* = 90 °C, 20 min
Detergents	Yes
For squeezing	Δ*p* = 40–60 bar, 10 s
For drying (passing through calenderers)	*T* = 200 °C, 15 s

**Table 4:** Performance of passive RFID UHF tag models and number of wash limit cycles.

Tag Model	Materials for UHF Antenna	Average Reading Speed (Readings/s)	Average Number of Successful Readings (%)	Average Number of Wash Limit Cycles
M1	Electroconductive paint	30	85	31
M2	Copper plated conductor with silver	37	97	20
M3	Electroconductive textile thread	35	95	30
M4	Multi-filament stainless steel wire	40	100	400

**Table 5:** Description of UHF RFID tag variants.

Washable Tag Variant	Dimensions		View of UHF RFID Tag	UHF Antenna with
	*L* [mm]	*l* [mm]		
V1-Switzerland [41]	70	15	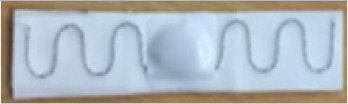	single loop
V2-Turkey [42]	70	15	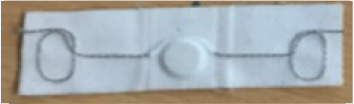	single loop
V3-France [43]	57	20	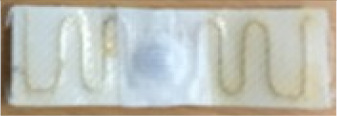	single loop
V4-China [44]	62	10	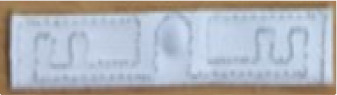	single loop
V5-Dac100	57	13	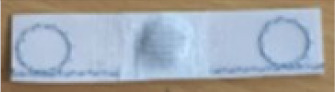	double loop

**Table 6:** The threshold power *P_th_* and the read range *d_r_* at 900 MHz for tested UHF RFID tag variants.

Washable Tag Variant	*P_th_* (dBm)	*d_r_* (m)
V1-Switzerland [41]	6.8	7.2
V2-Turkey [42]	7.8	5.7
V3-France [43]	9.7	5.2
V4-China [44]	12.0	4.3
V5-Dac100	8.0	5.9

## References

[1] Arshad R., Zahoor S., Shah M.A., Wahid A., Yu H. (2017). Green IoT: An investigation on energy saving practices for 2020 and beyond. IEEE Access.

[2] Alexandru A., Tudora E., Bica O. (2010). Use of RFID technology for identification, traceability monitoring and the checking of product authenticity. World Acad. Sci. Eng. Technol. Int. Sch. Sci. Res. Innov..

[3] Dobkin D. (2008). The RF in RFID Passive UHF RFID in Practice.

[4] Rao K.V.S., Nikitin P.V., Lam S.F. (2005). Antenna design for UHF RFID tags: A review and a practical application. IEEE Trans. Antennas Propag..

[5] Finkenzeller K. (2010). RFID Handbook-Fundamentals and Application in Contactless Smart Cards, Radio Frequency Identification and Near-Field Communication.

[6] Colella R., Catarinucci R.L., Tarricone L. Improved RFID Tag Characterization System: Use Case in the IoT Arena. Proceedings of the RFID-TA.

[7] Saygin C. (2007). Adaptive inventory management using RFID data. Int. J. Adv. Manuf. Technol..

[8] Abad E., Palacio F., Nuin M., González de Zárate A., Juarros A., Gómez J.M., Marco S. (2009). RFID smart tag for traceability and cold chain monitoring of foods: Demonstration in an intercontinental fresh fish logistic chain. J. Food Eng..

[9] Todorovic V., Neag M., Lazarevic M. (2014). On the usage of RFID tags for tracking and monitoring of shipped perishable goods. Procedia Eng..

[10] Gaynor M., Waterman J. (2016). Design framework for sensors and RFID tags with healthcare applications. Health Policy Technol..

[11] Moraru A., Helerea E., Ursachi C., Călin M.D. RFID system with passive RFID tags for textiles. Proceedings of the ATEE.

[12] Moraru A., Helerea E., Ursachi C. Passive RFID tags for textile items—Requirements and solutions. Proceedings of the ISFEE.

[13] Konovalenko I., Ludwig A. (2019). Event processing in supply chain management—The status quo and research outlook. Comput. Ind..

[14] Björninen T., Virkki J., Sydänheimo L., Ukkonen L. Impact of recurrent washing on the performance of electro-textile UHF RFID tags. Proceedings of the RFID-TA.

[15] Moraru A., Helerea E., Ursachi C. (2018). RFID Passive Tags for Harsh Industrial Environments. Bull. Transilv. Univ. BraşovSer. I Eng. Sci..

[16] Virkki J., Björninen T., Kellomäki T., Merilampi S., Chan Y.C. (2014). Reliability of washable wearable screen printed UHF RFID tags. Microelectron. Reliab..

[17] Janeczek K., Kozioł G., Serzysko T., Jakubowska M. Investigation of RFID tag antennas printed on flexible substrates using two types of conductive pastes. Proceedings of the ESTC.

[18] Toivonen M., Björninen T., Sydänheimo L., Ukkonen L., Rahmat-Samii Y. (2013). Impact of moisture and washing on the performance of embroidered UHF RFID tags. IEEE Antennas Wirel. Propag. Lett..

[19] Guibert M., Massicart A., Chen X., He H., Torres J., Ukkonen L., Virkki J. Washing reliability of painted, embroidered, and electro-textile wearable RFID tags. Proceedings of the PIERS—FALL.

[20] Simorangkir R.B.V.B., Le D., Björninen T., Sayem A.S.M., Zhadobov M., Sauleau R. (2019). Washing Durability of PDMS-Conductive Fabric Composite: Realizing Washable UHF RFID Tags. IEEE Antennas Wirel. Propag. Lett..

[21] Virkki J., Bjorninen T., Merilampi S., Sydanheimo L., Ukkonen L. (2015). The effects of recurrent stretching on the performance of electro-textile and screen-printed ultra-high-frequency radio-frequency identification tags. Text. Res. J..

[22] Lahokallio S., Saarinen-Pulli K., Frisk L. (2015). Effect of different test profiles of temperatures cycling tests on the reliability of RFID tags. Microelectron. Reliab..

[23] Salman K.N., Ismail A., Raja Abdullah R.S.A., Saeedi T. (2017). Coplanar UHF RFID tag antenna with U-shaped inductively coupled feed for metallic applications. PLoS ONE.

[24] Saarinen-Pulli K., Lahokallio S., Frisk L. (2016). Effects of different anisotropic conductive adhesives on the reliability of UHV RFID tag. Int. J. Adhes. Adhes..

[25] Janeczek K. (2017). Reliability analysis of UHF RFID tag under long term mechanical cycles. Microelectron. Reliab..

[26] Moraru A., Ursachi C., Helerea E. Near and far field measurements in industrial environment of passive UHF RFID tags. Proceedings of the ATEE.

[27] The Alien Technology for RFID, Alien Technology, 845 Embedded Way San Jose, CA. www.alientechnology.com.

[28] Kutluhan U. (2013). The Enhanced Antenna Structure for RFID Tags.

[29] Naoya K., Hidehiko K., Noriyuki O., Kouichi U. (2009). RFID Tag. U.S. Patent.

[30] Pachoud D., Stegmaier P. (2012). Textile Item Identification Tag.

[31] Arora K., Mallinson H., Kulkarni A., Brusey J., McFarlane D. The practical feasibility of using RFID in a metal environment. Proceedings of the 2007 IEEE Wireless Communications and Networking Conference.

[32] Griffin J.D., Durgin G.D., Haldi A., Kippelen B. (2006). RF tag antenna performance on various materials using radio link budgets. IEEE Antennas Wirel. Propag. Lett..

[33] Rokunuzzaman M., Islam M.T., Rowe W.S.T., Kibria S., Singh M.J., Misran N. (2016). Design of a Miniaturized Meandered Line Antenna for UHF RFID Tags. PLoS ONE.

[34] Moraru A., Ursachi C. (2018). Radio-Frequency Identification Transponder for Aggressive Environments.

[35] Marrocco G. (2008). Advanced UHF RFID tag antenna design. IEEE Antennas Propag. Mag..

[36] VMware and Integrated Environments, Circuits. http://www.circuits.dk/calculator_%20planar_coil_inductor.htm.

[37] Bansal A., Sharma S., Khanna R. A spiral shaped loop fed high read range compact tag antenna for UHF RFID applications. Proceedings of the RFID-TA.

[38] Moraru A., Ursachi C. (2020). RFID Tag for Harsh Environment Inductively Coupled in Double Loop. U.S. Patent.

[39] Coil Inductance. https://www.eeweb.com/tools/coil-inductance.

[40] Tagformance pro, Voyantic Ltd., Espoo, Finland. http://voyantic.com/Tagformance_pro.

[41] Textile ID Datamars. https://textile-id.com/products-to-identify-and-track-every-kind-%20of-textile/#15725%2041468213-7b13fc6c-2d51.

[42] USTEK RFID Solutions. https://ustek-rfid.com/products/uhf-rfid-tags/.

[43] Fenotag. https://fenotag.com/products/.

[44] Invengo Technologies SARL. https://www.invengo-textile.com/%20uhf-laundry-tags-and-hardware/.

